# Quick-EXAFS setup at the SuperXAS beamline for *in situ* X-ray absorption spectroscopy with 10 ms time resolution

**DOI:** 10.1107/S1600577515018007

**Published:** 2016-01-01

**Authors:** Oliver Müller, Maarten Nachtegaal, Justus Just, Dirk Lützenkirchen-Hecht, Ronald Frahm

**Affiliations:** a Department of Physics, University of Wuppertal, Gaußstraße 20, 42119 Wuppertal, Germany; b Paul Scherrer Institute, 5232 Villigen, Switzerland; c Helmholtz-Zentrum Berlin für Materialien und Energie, Hahn-Meitner-Platz 1, 14109 Berlin, Germany

**Keywords:** QEXAFS, XAS, X-ray monochromator, time-resolved spectroscopy, synchrotron instrumentation

## Abstract

The quick-EXAFS (QEXAFS) method adds time resolution to X-ray absorption spectroscopy (XAS) and allows dynamic structural changes to be followed. A completely new QEXAFS setup consisting of monochromator, detectors and data acquisition system is presented, as installed at the SuperXAS bending-magnet beamline at the Swiss Light Source (Paul Scherrer Institute, Switzerland). The monochromator uses Si(111) and Si(311) channel-cut crystals mounted on one crystal stage, and remote exchange allows an energy range from 4.0 keV to 32 keV to be covered. The spectral scan range can be electronically adjusted up to several keV to cover multiple absorption edges in one scan. The determination of the Bragg angle close to the position of the crystals allows high-accuracy measurements. Absorption spectra can be acquired with fast gridded ionization chambers at oscillation frequencies of up to 50 Hz resulting in a time resolution of 10 ms, using both scan directions of each oscillation period. The carefully developed low-noise detector system yields high-quality absorption data. The unique setup allows both state-of-the-art QEXAFS and stable step-scan operation without the need to exchange whole monochromators. The long-term stability of the Bragg angle was investigated and absorption spectra of reference materials as well as of a fast chemical reaction demonstrate the overall capabilities of the new setup.

## Abstract 2

A new quick-scanning EXAFS (QEXAFS) monochromator, ionization chambers and data acquisition system have been developed and installed at the SuperXAS beamline at the Swiss Light Source to reach a temporal resolution of 10 ms.

## 1. Introduction   

Quick-EXAFS (QEXAFS) enables time-resolved X-ray absorption fine structure (XAFS) spectroscopy down to the millisecond time scale (Frahm, 1988 ▸, 1989 ▸; Stötzel *et al.*, 2010*a* ▸; Fonda *et al.*, 2012 ▸; Nonaka *et al.*, 2012 ▸). The X-ray absorption near-edge structure (XANES) and the extended X-ray absorption fine structure (EXAFS) regions, which give a detailed view of the local electronic and geometric structure of matter (Lee *et al.*, 1981 ▸; Rehr & Albers, 2000 ▸; Ankudinov *et al.*, 2002 ▸), can be measured with high repetition rates in real time. The unique combination of element sensitivity, time resolution and the high penetration depth of hard X-rays makes QEXAFS an invaluable tool for *in situ* investigations of chemical reactions and short-lived intermediates. The analyses of QEXAFS data sets give the reaction rate of a specific site. When coupled to the global reaction rate it can be determined whether that specific site is involved in the rate-limiting step (Bravo-Suárez *et al.*, 2008 ▸). Also, static sample systems can benefit from fast repetitive measurements, *e.g.* for screening a large number of samples in a batch, or radiation-sensitive samples can be measured as a function of radiation dosage providing a method to obtain a spectrum before the damage becomes critical. This is an important issue especially for biological samples (Yano *et al.*, 2005 ▸). Hence, applications cover a broad field of various disciplines from physics, chemistry, geology, materials science and others (Doronkin *et al.*, 2014 ▸; Gänzler *et al.*, 2015 ▸; Ohyama *et al.*, 2011 ▸; Stötzel *et al.*, 2010*b* ▸).

QEXAFS is basically a straightforward evolution of conventional step-scanning measurements and is therefore directly applicable to the same samples, the same sample environments and the same detection schemes. This also includes de-excitation processes such as fluorescence or total electron yield, which are important for dilute samples or surfaces.

Other time-resolved XAS techniques include energy-dispersive X-ray absorption spectroscopy (EDXAS) (Pascarelli & Mathon, 2010 ▸) and high-energy resolution off-resonant spectroscopy (HEROS) (Błachucki *et al.*, 2014 ▸). HEROS is an alternative method to obtain XANES spectra in fluorescence geometry. Measured spectra are free of self-absorption effects and exhibit a high energy resolution. Using this method, the sample is irradiated with a fixed monochromatic photon energy tuned below the absorption edge of interest. X-ray fluorescence emitted by the sample is measured by means of a dispersive spectrometer and transformed into XAS spectra. This method is intrinsically capable of single-shot measurements (Szlachetko *et al.*, 2014 ▸). However, due to the strong signal dampening, HEROS does not allow time-resolved measurements of the full EXAFS region.

The QEXAFS technique uses a dedicated X-ray monochromator to enable rapid and repetitive energy scans by oscillating the monochromator crystals. The photon energy is increased and decreased within one cycle and thus the acquisition rate of absorption spectra is twice the mechanical oscillation frequency.

The monochromator presented here allows remote adjustment of the oscillation frequency and spectral range. It is equipped with two different channel-cut crystals, Si(111) and Si(311), to extend the accessible X-ray energy range. These can be exchanged remotely without the need to open the vacuum chamber. Major improvements of ionization chambers led to the gridded ionization chamber design (Müller *et al.*, 2013 ▸), which is more than two orders of magnitude faster than common parallel-plate ionization chambers. This development is the key component to reach temporal resolutions below 100 ms. The current setup enables acquisition rates of up to 100 spectra s^−1^, reaching a time resolution of 10 ms.

## 2. Layout and performance of the QEXAFS monochromator at the SuperXAS beamline   

### 2.1. Description of the SuperXAS beamline   

**Figure 1:**
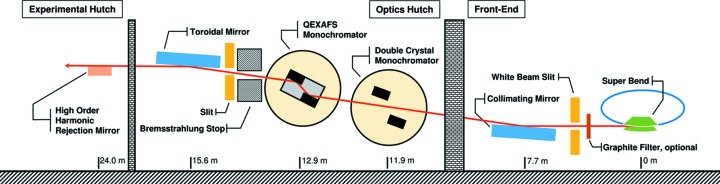
Schematic view of the optical components installed at the SuperXAS beamline.

The SuperXAS beamline is dedicated to time-resolved XAS and X-ray emission spectroscopy (XES). It is located at the Swiss Light Source (SLS) at the Paul Scherrer Institute (Villigen, Switzerland). The synchrotron radiation facility is operated in top-up mode with 400 mA at 2.4 GeV electron energy. Fig. 1 ▸ shows a schematic overview of the optical components of the beamline. A Super Bend magnet with a critical energy of 11.1 keV delivers X-rays over a broad spectral range, covering roughly 4 keV to 32 keV with a high flux of 10^11^ photons s^−1^ to 10^12^ photons s^−1^ in the monochromatized beam. The first optical component after the Super Bend magnet is a collimating mirror, which collects the radiation of the bending magnet and shifts its source point to infinity, thus eliminating the beam divergence in the vertical direction to preserve the energy resolution of the monochromator. At the same time this mirror cuts off the high energy part of the emitted spectrum thereby reducing the heat load onto the first monochromator crystal and partially removing higher harmonics from the monochromatized beam. The suppression of higher-order harmonics can be further improved by a dedicated mirror downstream in the experimental hutch. This promotes the usage of channel-cut crystals which are, due to their high intrinsic stability, favorably used in QEXAFS monochromators, but lack a possibility to suppress higher harmonics by detuning. The low-energy part of the emitted spectrum is removed by graphite filters of various thicknesses. The collimating mirror is followed by a conventional fixed-exit double-crystal monochromator and the QEXAFS monochromator. The new QEXAFS monochromator was installed and commissioned at the SuperXAS beamline in January 2015 and has been available for users since then. It replaces the former QEXAFS monochromator (Frahm *et al.*, 2010 ▸).

**Figure 2:**
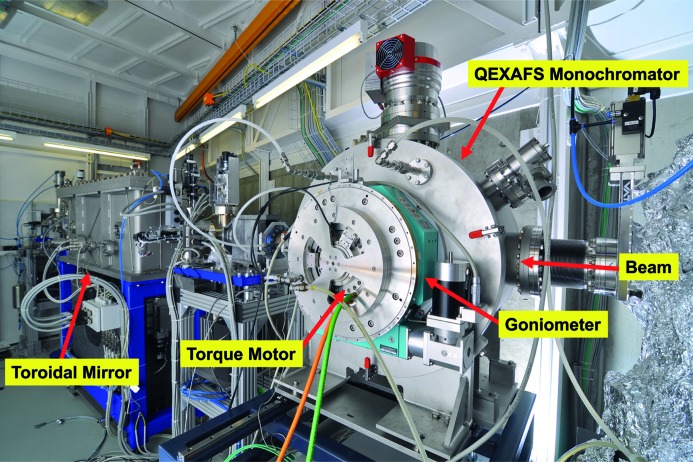
Photograph of the interior of the optics hutch of the SuperXAS beamline. The collimated beam enters from the right. The QEXAFS monochromator is recognizable by the turquois goniometer in front of the vacuum chamber of the monochromator.

The second downstream mirror is a toroidal mirror used to focus the monochromatic X-ray beam onto the sample, down to typically 100 µm × 100 µm. The focusing optics also maintains the beam position on the sample within 50 µm, since it is tolerant to small vertical beam movements which occur downstream of the monochromator during an energy scan using a channel-cut crystal. Different coatings are available to change the cut-off energy and to alter the reflective properties of the mirrors. Both mirrors exhibit stripes of platinum and rhodium next to each other, while the collimating mirror also offers a bare polished silicon surface. A view of the interior of the optics hutch including the new QEXAFS monochromator is shown in Fig. 2 ▸.

### 2.2. The QEXAFS monochromator   

The QEXAFS monochromator needs to facilitate fast and continuous energy scans with a high repetition accuracy. This is realised by a smooth oscillation of the monochromator crystals, altering the Bragg angle and thus the reflected energy continuously. The crystal stage is driven by a direct-drive torque motor, which is mounted inside a larger goniometer. Both are located outside on the atmospheric side of the vacuum chamber. Rotations are fed to the inside by a ferrofluidic sealed rotary feedthrough. The torque motor is used to drive the oscillatory movement of the crystals during QEXAFS operation, while the outer goniometer facilitates precise alignment of the center angle of the crystals and also enables step-scanning capabilities. The Bragg angle is measured by a high-resolution in-vacuum optical incremental encoder. An auxiliary in-vacuum absolute encoder is added to the same axis to simplify energy calibration.

Two different channel-cut crystals, Si(111) and Si(311), are mounted side by side on the oscillatory stage to cover the spectral range delivered by the bending magnet. The crystal is selected by a horizontal movement of the vacuum chamber, which is mounted on a horizontal and a vertical linear translation stage, perpendicular to the incident beam. Flexible bellows connect the monochromator to the beamline and allow a total movement of ±35 mm. A comprehensive description of the monochromator mechanics and its performance can be found in Müller *et al.* (2015*a* ▸).

### 2.3. Capabilities and performance of the QEXAFS monochromator   

**Figure 3:**
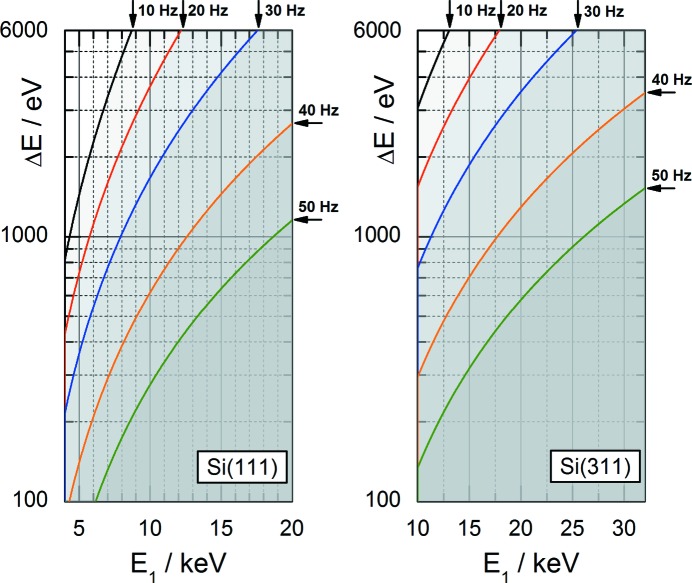
The solid curves show the accessible spectral range from 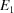 to 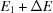 of a QEXAFS measurement at constant oscillation frequency using Si(111) and Si(311) crystals. QEXAFS measurements are possible within the greyish areas beneath the solid curves.

As in conventional XAS measurements, the spectral range of QEXAFS spectra is given by the covered Bragg angle range. It is thus defined by the amplitude and center position of the crystal oscillation, which can be selected using the goniometer. The maximum amplitude is limited by the torque of the direct-drive motor and decreases with higher oscillation frequencies. The two plots of Fig. 3 ▸ indicate the resulting spectral range 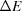 as a function of the start energy 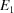 based on the largest possible amplitude at a certain oscillation frequency. Consequently, the grey area below any solid curve represents the accessible spectral range at this frequency. It can be seen that the scan range can be quite large, for instance to achieve simultaneous acquisition of several absorption edges.

### 2.4. Long-term stability   

**Figure 4:**
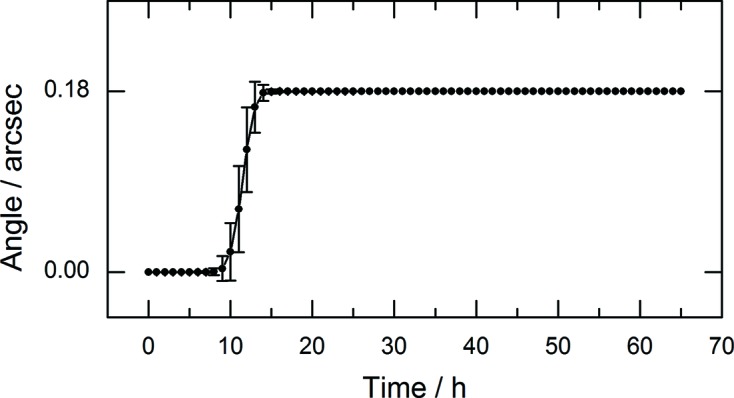
Deflection of the Bragg angle in the non-QEXAFS mode recorded over 65 h. The black dots display the mean angular position averaged over 1 h and the error bars indicate their standard deviation.

The long-term stability of the Bragg angle is of particular importance in the non-QEXAFS mode. This mode is required for step-scanning techniques, such as conventional XAS or XES methods and fixed-energy experiments, such as HEROS or laser pump/X-ray probe experiments. To realise solid stability, the rotor of the torque motor can be mechanically locked from the atmospheric side by a metal clamp. In this case, the goniometer still facilitates rotations of the crystals over the entire angular range and the in-vacuum angular encoder can still be used to read the current Bragg angle. The stability was verified by continuous sampling of the in-vacuum encoder output at 10 kHz over 65 h. Over the entire time, the encoder readings toggled only between two adjacent discrete values separated by its angular resolution of 0.18 arcsec. The mean angular position averaged over 1 h is plotted over time in Fig. 4 ▸. The angular position is static within adjacent values and there is no real drift. Taking into account a typical Darwin width (FWHM) of, for example, 6.6 arcsec at 9 keV for Si(111) or 1.5 arcsec at 16 keV for Si(311), the Bragg angle can be considered stable over the full observation period, for any practical application. The theoretical energy deviations caused by an angular change of 0.18 arcsec amount to 0.03 eV or 0.06 eV, respectively, for the mentioned examples.

## 3. The QEXAFS data acquisition system   

During a QEXAFS scan the X-ray intensities change quickly. In particular the transmitted intensity runs through fast changes at the absorption edge. Therefore, special demands concerning the response times of the detectors, *i.e.* ionization chambers, and the data acquisition system (DAQ) need to be taken into account. A good measurement implies that each significant data point of a spectrum is measured completely independent of the previous one. Based on the intrinsic energy resolution of the employed crystals, 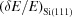 = 1.4 × 10^−4^ and 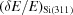 = 0.3 × 10^−4^, and the mean scan rate of the measurement, 2π*f*_Osc_Δ*E*, where 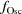 is the mechanical oscillation frequency of the monochromator and 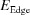 is the absorption edge energy, a time frame per data point 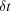 can be determined:
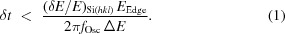
This time frame can be understood as the upper limit for the required response time of the entire detector system, including ionization chambers, current amplifiers and analog-to-digital-converters. This leads to crucial consequences for sub-second QEXAFS measurements. For instance, a measurement with 30 Hz oscillation frequency at the Cu *K*-edge at 8.979 keV covering a spectral range of 1.1 keV including a short pre-edge region requires a time frame of less than 19 µs per data point according to equation (1). This is much shorter than the response time of parallel-plate ionization chambers, which typically is in the region of several 100 µs at best (Müller *et al.*, 2013 ▸; Knoll, 2000 ▸). This is due to the underlying working principle of parallel-plate ionization chambers, whose response is governed by the drift motion of all charged particles created in consequence of the photoelectric absorption of incident X-ray photons. During this process mainly positive ions and electrons are released. The comparatively slow ion drift velocity is mainly responsible for the length of the response time. Although this drift velocity can be increased with a higher voltage across the electrodes or a shorter distance between them, a response time in the low microsecond regime is not feasible due to the limited dielectric strength of the typical filling gases, such as argon or nitrogen. A solution to this problem is the use of gridded ionization chambers which suppress the influence of the ion movement almost completely (Müller *et al.*, 2013 ▸). Since in those detectors essentially only electrons contribute to the ionization current, significantly faster response times are achievable. The actual response time of the employed gridded ionization chamber including the current amplifier at a gain of 10^6^ V A^−1^ was determined by means of a fast X-ray chopper (Müller *et al.*, 2015*b* ▸) and amounts to less than 5 µs, which is well matched for the pursued measurements.

### 3.1. The data acquisition system   

The data acquisition system (DAQ) used to record QEXAFS spectra and to control the monochromator is based on the National Instruments PXIe-6366 multifunctional data acquisition board. This board is installed in a chassis (NI PXIe-1073) next to a glass fiber bridge (NI PXIe-PCIe8375) which connects to a host PC located in the control hutch and ensures a ground-free connection. The chassis is placed on the experimental table close to the current amplifiers and ionization chambers. The DAQ provides several analog-to-digital-converters (ADCs), digital-to-analog-converters (DACs) and general purpose digital input output pins (GPIOs). The output voltages of the current amplifiers were digitized simultaneously by means of the ADCs (16 bit, ±10 V) provided by the DAQ. Sinusoidal control voltages, which are required to drive the power supplies of the torque motor, were generated with two DAC channels (16 bit, ±10 V). The GPIOs were used to read the incremental quadrature output of the angular encoder.

**Figure 5:**
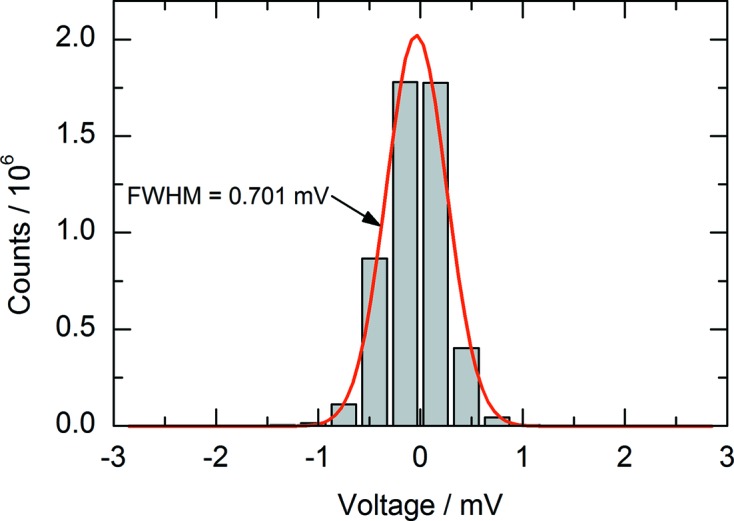
Noise level sampled at 2 MHz with powered ionization chambers and current amplifiers at a gain of 10^6^ V A^−1^, but without X-ray beam. The FWHM of 5 × 10^6^ samples amounts to 0.701 mV and is centered around −0.039 mV.

The detector output voltages are digitized by the ADCs running with their highest sampling frequency of 2 MHz so that the QEXAFS measurements are strongly oversampled. The oversampling allows high frequencies of the noise spectrum originating from the detection system and the ADC itself to be resolved. Subsequent digital low-pass filtering can be performed to improve the signal-to-noise ratio. To minimize the background noise level, the DAQ was carefully set up, particularly with regard to cable length, possible ground loops and mechanical vibrations, *e.g.* caused by nearby vacuum pumps. Table-top high-voltage power supplies (ISEG, THQ DPS 30-405-24-5) were chosen to operate the ionization chambers, due to the low ripple of less than 3 mV at 3 keV output voltage. This is crucial for minimizing noise in the output signals of these detectors. The frequency distribution of single bits occurring in an unfiltered raw measurement is shown in Fig. 5 ▸. The signal strength of the gridded ionization chamber connected to the current amplifier was sampled for 2.5 s at 2 MHz while the X-ray beam was shut off. The FWHM of the ground noise level amounts to 0.701 mV. This results in a signal-to-noise ratio of better than 10^3^, assuming a typical signal level of a few Volts during the measurements.

#### 3.1.1. Synchronization   

**Figure 6:**
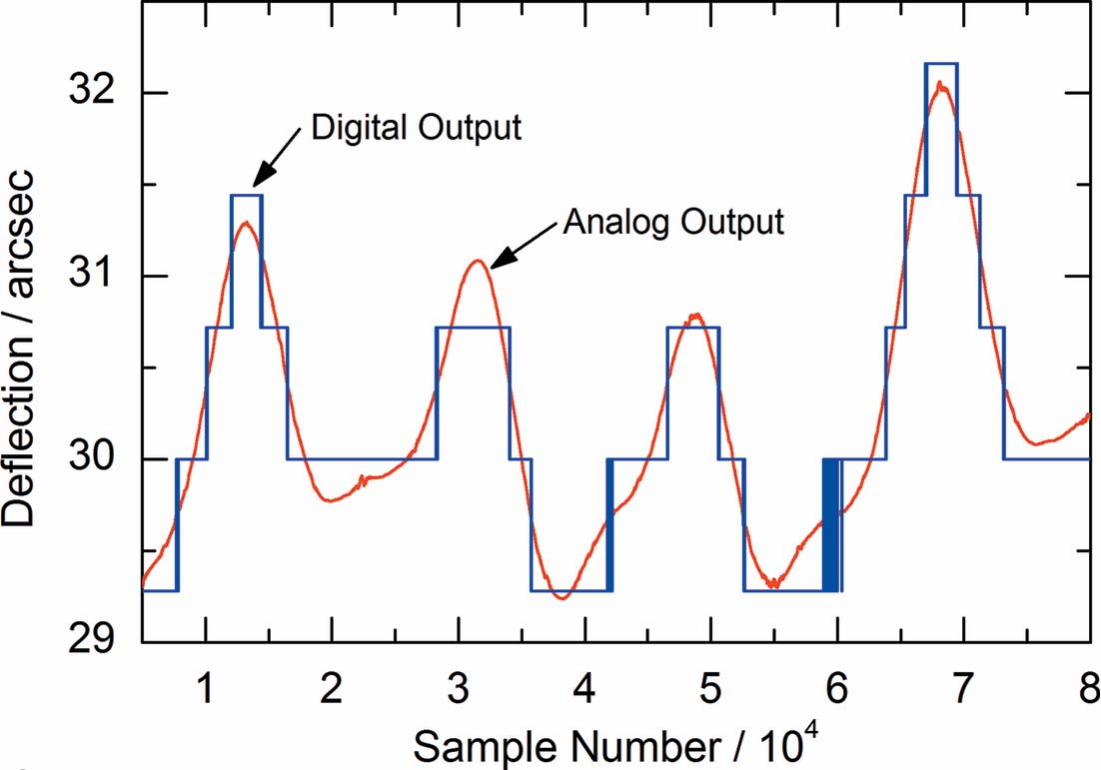
Digital and analog incremental encoder output of random angular noise simultaneously sampled at 2 MHz to verify the synchronization of the analog and digital part of the data acquisition system.

All devices of the DAQ are simultaneously triggered by a single on-board reference clock. Proper synchronization was verified by simultaneous measurements of correlated analog and digital signals. The interpolator (Renishaw DOP0400A50A) of the angular encoder provided suitable signals, since it outputs the angular information in the form of an analog sin/cos and a digital quadrature signal. Fig. 6 ▸ shows random angular noise which was recorded during relaxation, after the crystal stage of the monochromator has been slightly displaced. The analog and digital signals overlap without a noticeable phase-shift, which confirms the excellent synchronization.

## 4. QEXAFS applications and measurements   

Measurements to illustrate the capabilities and the potential of the new QEXAFS setup were performed subsequent to the commissioning phase. All absorption measurements were obtained in transmission geometry. Fast gridded ionization chambers and home-made current amplifiers were used to measure the incident and transmitted X-ray intensities.

### 4.1. Quick-XANES and Quick-EXAFS at the Cu *K*-edge   

The QEXAFS capabilities were tested by XANES and EXAFS measurements of a copper metal foil at the Cu *K*-edge at 8979 eV using the Si(111) crystal of the monochromator.

**Figure 7:**
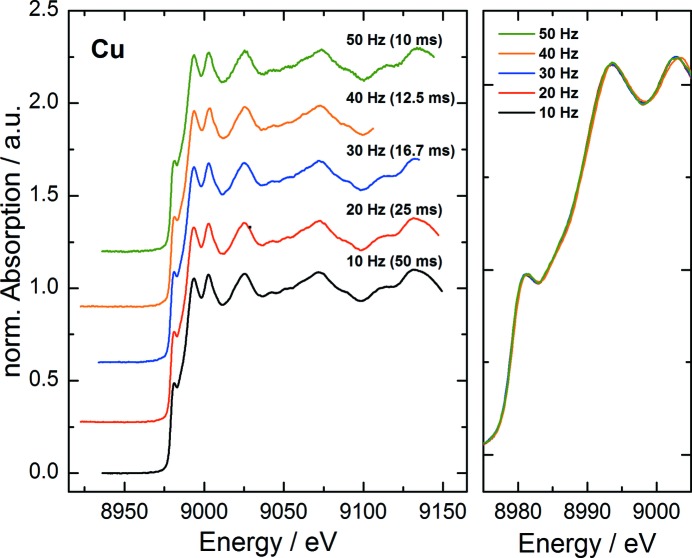
Single ‘down’ XANES spectra of a Cu metal foil at the Cu *K*-edge at various oscillation frequencies ranging from 10 Hz to 50 Hz.

The XANES of metallic copper shows a sharp distinct edge feature in the form of a small edge peak. Due to its sharpness the measurement is very sensitive to mechanical instabilities of the monochromator, its energy resolution and the bandwidth limitations of the detector system. It is therefore quite challenging to reproduce the near-edge structure undisturbed at very high scan rates. To verify the achievable precision with the presented setup at the SuperXAS beamline, spectra have been acquired with different acquisition rates ranging from 20 to 100 spectra per second. The measured spectra are typically referred to either ‘up’ or ‘down’ depending on the energy scan direction. Fig. 7 ▸ shows single XANES ‘down’ spectra. The right-hand plot displays the same spectra as displayed in the left-hand plot, but without vertical offset. With this representation it becomes visible that all measured ‘down’ spectra, independent of the acquisition rate, are in fact identical, proving the excellent accuracy and reproducibility of the complete data acquisition system.

**Figure 8:**
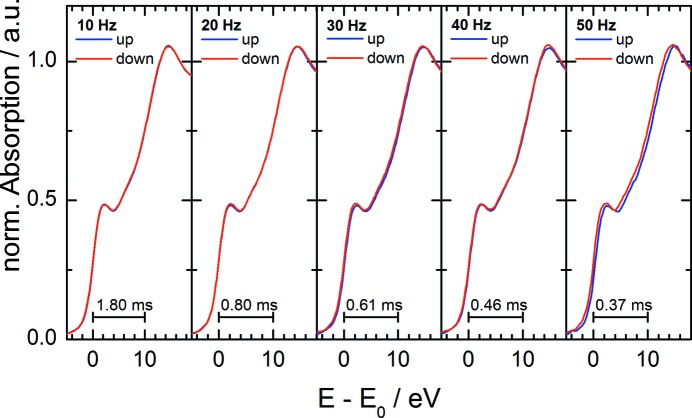
Consecutive ‘up’ and ‘down’ XANES spectra of a Cu metal foil at the Cu *K*-edge measured at different oscillation frequencies.

Consecutive ‘up’ and ‘down’ spectra are compared in Fig. 8 ▸. The individual plots show the same section of the spectrum around the absorption edge. Consecutive spectra are plotted on top of each other to make even small deviations visible. Up to oscillation frequencies of 40 Hz no differences depending on the scan direction are noticeable. At 50 Hz a small shift between the ‘up’ and ‘down’ measured spectrum appears, amounting to approximately 0.3 eV at the edge position. This shift is assumed to be caused by the limited bandwidth of the detectors, since the shift is not constant throughout the spectrum but depends on the local rate of change of the spectra. It is found that this deviation is the same in every measured ‘up’ spectrum. Therefore both spectra, *i.e.* ‘up’ and ‘down’, can be used for proper data evaluation, implying that a true temporal resolution of 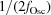 at frequencies of up to 50 Hz can be reached at least for XANES measurements with this setup.

**Figure 9:**
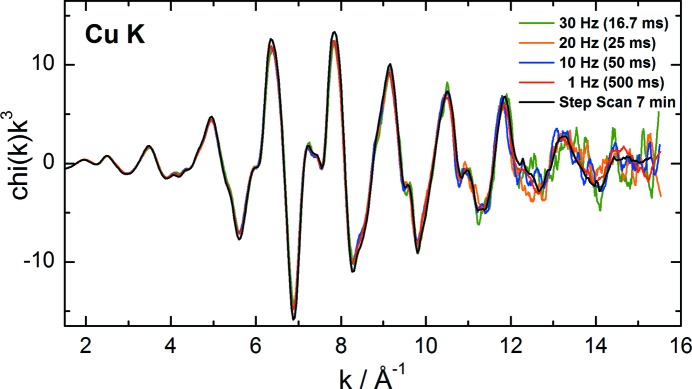
Extracted *k* ^3^-weighted EXAFS of a Cu metal foil measured at the Cu *K*-edge with various monochromator oscillation frequencies, and in step-scanning mode.

To investigate the capabilities of the beamline for fast EXAFS measurements, the *K*-edge EXAFS spectra of metallic copper have also been measured at different oscillation frequencies between 1 Hz and 30 Hz. The angular range was set to 1.4° to cover an energy range of about 1.1 keV. The extracted fine structures of single spectra are *k* ^3^-weighted and plotted on top of each other in Fig. 9 ▸. The measurements show that, although the noise increases with higher acquisition frequencies, meaningful EXAFS up to 14 Å^−1^ can be obtained at 30 Hz oscillation frequency. A single EXAFS spectrum recorded at 30 Hz is measured in less than 17 ms.

### 4.2. Real-time observation of nanocrystal formation simultaneously at the Cu and Zn *K*-edges   

To demonstrate the capabilities of the QEXAFS setup, results of a fast chemical reaction are presented. Here, the formation mechanism of multinary Cu_2_ZnSnS_4_ (CZTS) nanocrystals has been investigated in real time. CZTS is a potential candidate for absorber layers in thin-film solar cells which can be synthesized in nano-crystalline form by a hot-injection one-pot synthesis (Polizzotti *et al.*, 2013 ▸; Shin *et al.*, 2013 ▸; Singh *et al.*, 2012 ▸; Guo *et al.*, 2010 ▸). Of particular interest during growth is the incorporation kinetics of different atomic species which have been found to be subsequently included into the nanocrystals (Coughlan & Ryan, 2015 ▸).

**Figure 10:**
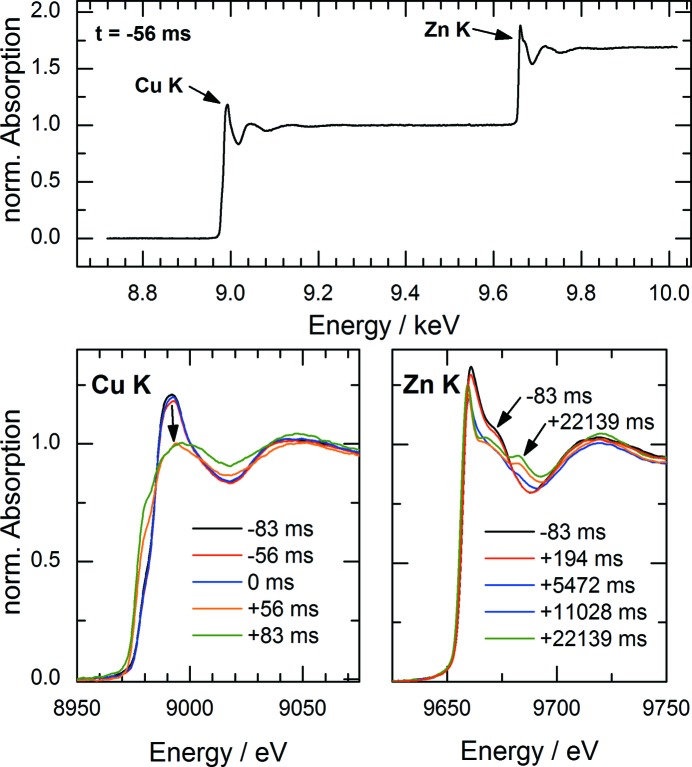
Measurement of Cu and Zn *K*-edges acquired with 36 spectra per second. The reaction is triggered at *t* = 0 s by fast injection of a chalcogene source.

The nanocrystal formation from solved metal salts of Cu, Zn and Sn was investigated using an *in situ* liquid chemical reactor for transmission-mode QEXAFS. The reactor is capable of triggering the chemical reaction by fast injection of a chalcogen source within less than 20 ms. Details of the experimental setup as well as a detailed discussion of the achieved results will be published elsewhere. The absorption spectra of the Cu *K*- and Zn *K*-edges have been simultaneously acquired with an oscillation frequency of 18 Hz yielding a temporal resolution of 27.8 ms. The measured energy range of about 1.3 keV covering both *K*-edges is shown in Fig. 10 ▸ (top) for one specific spectrum of the solved precursors 56 ms before injection. The reaction is triggered at *t* = 0 s by injection of a reactant. The injection process is quite fast, but causes turbulences and thus inhomogeneities which cause the first spectrum after injection to be unusable for EXAFS analysis.

For all subsequent spectra measured during the course of the reaction, it is possible to extract the EXAFS of Cu as well as the XANES spectra of the Cu and the Zn *K*-edges with a high signal-to-noise ratio. In Fig. 10 ▸ (bottom) single spectra are shown around the trigger point of the reaction. It can be seen clearly that all spectra taken before the injection completely overlap, illustrating the stability and reproducibility of the measurement. The temporal resolution of the measurement given by the time per scan of 27.8 ms shows to be within a relevant time scale of the reaction kinetics as the Cu XANES changes substantially within a few measured spectra. In contrast, the reaction kinetics of the Zn precursor during incorporation into the nanocrystals exhibits a larger time scale of several seconds. The combination of measurements at the Cu and Zn *K*-edges in one scan enables a detailed understanding of the underlying reaction mechanism.

## 5. Conclusions and outlook   

A newly developed QEXAFS monochromator, ionization chambers and data acquisition system have been installed and commissioned at the SuperXAS beamline and are now available for users. The new setup replaces the former QEXAFS monochromator, which was successfully in operation since 2008. The monochromator uses a unique combination of a direct drive and an outer goniometer to drive the Bragg axis. This design combines both time-resolved QEXAFS and conventional step-scanning operation in one device at the beamline. Future upgrades will add further functions to the monochromator in the non-QEXAFS mode, to strengthen its value for conventional XAS and XES methods. The angular range of the crystal stage will be increased from 30° to more than 35° to access the iron *K*-edge using the Si(311) crystal. Due to its higher intrinsic energy resolution, XES experiments will substantially benefit from this upgrade.

The QEXAFS monochromator, in conjunction with the new data acquisition system and the gridded ionization chambers, enable a time resolution of 10 ms, yielding high data quality for XANES analysis. Cu *K*-edge EXAFS have been measured up to 15.5 Å^−1^ within 17 ms to illustrate the powerful Quick-EXAFS capabilities as well. The fast gridded ionization chambers allow using spectra of both scan directions directly with minor distortions only at the highest speed of 50 Hz. As shown, the spectral range can be extended to measure multiple absorption edges simultaneously on the millisecond time scale. First *in situ* experiments on real samples demonstrate the scientific potential of the setup to follow chemical reactions in real-time.
